# Intergenerational Transfer of Persistent Bacterial Communities in Female Nile Tilapia

**DOI:** 10.3389/fmicb.2022.879990

**Published:** 2022-05-17

**Authors:** Yousri Abdelhafiz, Jorge M. O. Fernandes, Claudio Donati, Massimo Pindo, Viswanath Kiron

**Affiliations:** ^1^Faculty of Biosciences and Aquaculture, Nord University, Bodø, Norway; ^2^Unit of Computational Biology, Research and Innovation Centre, Fondazione Edmund Mach, San Michele all’Adige, Italy

**Keywords:** microbiome, buccal cavity, intestine, Nile tilapia, vertical microbe transfer, *Nocardioides*, *Propionibacterium*, *Sphingomonas*

## Abstract

Resident microbial communities that can support various host functions play a key role in their development and health. In fishes, microbial symbionts are vertically transferred from the parents to their progeny. Such transfer of microbes in mouthbrooder fish species has not been reported yet. Here, we employed Nile tilapia (*Oreochromis niloticus*) to investigate the vertical transmission of microbes across generations using a 16S rRNA amplicon sequencing approach, based on the presence of bacteria in different generations. Our analysis revealed that the core microbiome in the buccal cavity and posterior intestine of parents shapes the gut microbiome of the progeny across generations. We speculate that the route of this transmission is *via* the buccal cavity. The identified core microbiome bacteria, namely *Nocardioides, Propionibacterium*, and *Sphingomonas* have been reported to play an essential role in the health and development of offspring. These core microbiome members could have specific functions in fish, similar to mammals.

## Introduction

Microbial colonization and assemblage on various niches of hosts is a complex process, which is dependent on genetic as well as environmental background. Many studies have reported the significant role of these microbial communities in humans (Rackaityte and Lynch, 2020), fish (Legrand et al., 2020), and livestock (Cholewińska et al., 2021); they are composed of bacteria, fungi, and archaea (Berg et al., 2020). Different aspects of microbiota have been intensively studied to report valuable information about their influence on human health (Rackaityte and Lynch, 2020). Microbiota supports many functions to satisfy the nutritional needs of the host, mainly due to the ability of the microorganisms to produce vitamins (Nagy-Szakal et al., 2012) and valuable metabolites such as short chain fatty acids (García-Mantrana et al., 2019; Silva et al., 2020). In addition, host-associated microbes train and modulate the immune system to establish tolerance to commensal bacteria (Chai et al., 2014) as well as ward off invasive pathogens (Sylvain and Derome, 2017; Ferretti et al., 2018; Yukgehnaish et al., 2020; Cholewińska et al., 2021). It is noteworthy that early life food components can promote the colonization of specific microbes and their syntrophy with beneficial microbes (Kostopoulos et al., 2020). In fact, microbiome development during the early life of hosts helps in the intrinsic training of the immune functions and shaping of microbiome composition (Sylvain and Derome, 2017; Ferretti et al., 2018). Therefore, in the past years, scientists have been studying the vertical transfer of microbes from mother to infant (Ferretti et al., 2018; Rackaityte and Lynch, 2020) and from parents to progeny in animals (Sylvain and Derome, 2017; McGrath-Blaser et al., 2021; Mika et al., 2021). Microbe transfer in most organisms occurs in three ways: (i) vertical transfer of essential maternal microbes that aid host development at the early stage of life; (ii) horizontal transfer *via* ingestion of microbes from diet or surrounding environment to which host is exposed to; (iii) environmental transfer between conspecific organisms during social or sexual interaction (Leftwich et al., 2020). Nevertheless, the transfer routes vary across species. For example, in chicken, successive transfer of resident microbes takes place from the oviduct and cloaca to eggshell, egg white, and then the embryo (Lee et al., 2019). In livestock, microbes present in the birth canal colonize newborns (Estellé, 2019). In humans, microbes from the skin and vagina of mothers colonize different body sites of infants, and this type of vertical transmission continues through direct contact (Ferretti et al., 2018).

The mechanism of microbial transmission in aquatic animals differs from those of mammals. In most fish species, the early stage microbiome is shaped by the environment (Llewellyn et al., 2014). As the fish grows, the environmental influence will be overshadowed by other factors (Llewellyn et al., 2014). Atlantic salmon (*Salmo salar*) embryos were reported to have lower diversity compared to hatchlings, probably indicating the impact of factors other than the original determinant (Lokesh et al., 2019). In zebrafish (*Danio rerio*) larvae, horizontal transmission of microbial symbionts occurs from the surrounding environment (Stephens et al., 2016). In another fish model, discus (*Symphysodon aequifasciata*), also the larvae obtain their microbial symbionts *via* horizontal transmission from the surrounding water (Sylvain and Derome, 2017). However, during the fry stage of discus, vertical transmission prevails because parents feed their skin mucus to their offspring (Sylvain and Derome, 2017). Interestingly, in pipefish (*Syngnathus typhle*), specific bacteria from both parents shape the microbiota of the embryo because eggs are transferred from mother to paternal pouches (Beemelmanns et al., 2019). The little skate (*Leucoraja erinacea*) egg capsule holds a variety of bacteria, which will be transferred to offspring (Mika et al., 2021). To our knowledge there are no publications on the microbial transmission from a mouthbrooder to their offspring. Hence, we used Nile tilapia (*Oreochromis niloticus*) females as a model to understand the bacterial transfer from mother to offspring, and across generations employing the 16S rRNA amplicon sequencing technology, based on the presence of bacteria in different samples.

## Materials and Methods

### Ethics Statement

This study was performed under a license from the Norwegian Animal Research Authority (FOTS ID 10427). The experiment was conducted according to the guidelines for research using experimental animals; 3Rs principle, fish welfare and respect toward animals were given weightage while balancing between experimental procedures and benefits from the results.

### Experimental Fish

**FIGURE 1:**
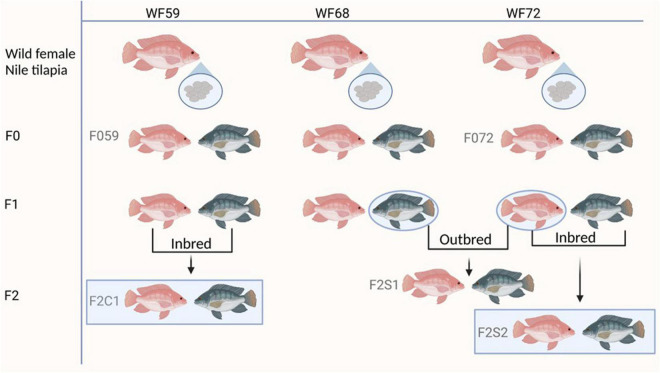
Breeding plan of the different generations of Nile tilapia. F0 generation: F059 and F072; F2 generation: F2C1, F2S1, and F2S2. Inbred groups (F2C1 and F2S2) and outbred (F2S1). WF59, W68, and WF72 are the wild mothers. The pink color represents female and gray male fish.

Nile tilapia for this study were produced from fertilized eggs that were being incubated by wild mouthbrooders, caught from Nile River, Luxor, Egypt (location GPS: 25°39′56″ N, 32°37′07″ E). The eggs were kept in a 60-L tank for 2 weeks. Water in these tanks was replaced with sterilized water every 2 days. The eggs hatched on day 5–6 after fertilization, and the larvae were transported to the research station of Nord University, Bodø, Norway. Juveniles obtained from different wild females were tagged and assigned as the base population or the first generation (F0). The second (F1) and third (F2) generations were obtained from the F0 generation. All fish generations were reared in a common garden in a recirculating aquaculture system for 8 months to avoid the influence of environmental confounding factors. Rearing conditions were: pH 7.6, oxygen saturation 100%, temperature 28°C, and photoperiod 11:13 dark:light. The experimental fish were fed *ad libitum* (0.15–0.8 mm) Amber Neptun pellets, Skretting, Norway (Konstantinidis et al., 2021). Figure 1 illustrates the breeding strategy to produce the experimental fish.

In the present study, we first examined the microbiota of the wild mouthbrooders (caught from River Nile, Egypt) from which their offspring (F0, maintained in Norway) were produced. To investigate the microbial transfer across generations, we employed two generations (F0 and F2) -from parent 1: F059 (F0), F2C1 (F2, fish from same parents); from parent 2: F072 (F0), F2S2 (F2, fish from same parents). Furthermore, to investigate differences in the microbial composition in F2, we compared the microbiota of two families (within F2; F2S1, F2S2 from F072 vs. F2C1 from F059) from the above mentioned F0 parents. The F1 generation [data published in Abdelhafiz et al. (2021a)] was not included in this study because we did not employ any microbial enrichment kits (Abdelhafiz et al., 2021a), which limits comparability between datasets.

### Sample Collection

Buccal cavity mucus and posterior intestine samples were collected from the wild-caught fish (*n* = 3) and transferred to cryotubes containing DNA/RNA shield (ZYMO Research Corp, Irvine, CA, United States). As for the samples from the fish reared in controlled conditions, they were collected from fish that were starved for 48 h. Buccal cavity and intestine samples from 20 fish (five fish in each group) were collected for the microbiota studies. Before collecting the samples, the fish were sacrificed by exposing them to an emulsion containing 12 mL of clove oil (Sigma Aldrich, MO, United States), 96% ethanol (1:10 v/v), and 10 L of water (Podgorniak et al., 2019). Mucus samples from the buccal cavity were taken using swabs (Copan Italia, Brescia, Italy), which were transferred to cryotubes and immediately frozen in liquid nitrogen. In addition, posterior intestine mucus samples were collected as described previously (Abdelhafiz et al., 2021a). The collected samples were stored at −80°C until further use.

### Microbial DNA Extraction and Library Preparation

DNA was extracted from both the mouth and posterior intestine using QIAamp DNA stool Mini Kit (Qiagen, Hilden, Germany) according to the manufacturer’s protocol. The collected samples were transferred to a 5 ml tube that contained 1.4 mm zirconium oxide beads (Cayman chemical, Ann Arbor, MI, United States). Then, 2 ml of InhibitEX buffer (Qiagen) was added to the tube. The extracted DNA was eluted in 75 μl ATE buffer. Thereafter, the quality and quantity of the extracted DNA were checked with NanoDrop spectrophotometer ND-8000 (Thermo Fisher Scientific Inc., Waltham, MA, United States).

Prior to library preparation, the extracted DNA from each sample was treated with REPLI-G kit (Qiagen) to enrich the microbial DNA. Library preparation and sequencing were performed as described previously (Abdelhafiz et al., 2021a).

### Data Processing and Analyses

Paired-end reads were truncated at 270 bp using VSEARCH (Rognes et al., 2016), and then processed using MICCA pipeline, V1.7.2 (Albanese et al., 2015). Sequences of paired-end reads with a minimum overlap length of 60 bp and a maximum mismatch of 20 bp were merged. Next, forward and reverse primers from the merged reads were trimmed off and the reads that did not contain the primers were discarded. The sequences with an expected error rate >0.75 were filtered out (Edgar and Flyvbjerg, 2015). Then the obtained reads were denoised using the “*de novo* unoise” method implemented in MICCA, which utilizes the UNOISE3 algorithm (Edgar, 2016). Thereafter, RDP classifier (Lan et al., 2012) was used to assign the taxonomic names of the representative bacterial amplicon sequence variants (ASVs). The sequences were aligned using the NAST (DeSantis et al., 2006) multiple sequence aligner, and a phylogenetic tree was prepared using the FastTree software available in the MICCA pipeline, as described previously (Abdelhafiz et al., 2021a,b). The downstream analyses were performed using the phyloseq package in R (McMurdie and Holmes, 2013).

### Statistical Analysis

To understand the differences in richness, evenness, and dominance of the bacterial communities across generations, we performed α-diversity analysis by calculating the Chao1 species richness, Shannon and Simpson diversities using estimate_richness function in *phyloseq* R package (version 1.38.0). Kruskal-Wallis rank sum test (R package *stats* version 4.1.2) and Dunn’s test (R package *rstatix* version 0.7.0) were used to check the differences between the study groups. On the other hand, to understand the dissimilarities in bacterial compositions, we performed β-diversity analysis using unweighted and weighted UniFrac distances (Lozupone and Knight, 2005). The differences were visualized by principal coordinates analysis (PCoA). After checking the dispersions within the data set of each generation using the beta.disper function in the R package *vegan* version 2.5-7, statistically significant differences between the groups were assessed using Permutational Multivariate Analysis of Variance Using Distance Matrices (Anderson, 2001), i.e., employing the adonis function implemented in the *vegan* R package version 2.5-7 (Oksanen et al., 2013). Furthermore, *post-hoc* test in *RVAideMemoire* R package (version 0.9-81) was employed to understand the differences between the groups. To detect the differentially abundant ASVs in the unrarefied data (Weiss et al., 2017), we used the R package *DESeq2* version 1.34.0 (Love et al., 2014). The core microbiome analysis was performed using *microbiome* (version 1.16.0) and *microbiomeutilities* (version 1.00.16) packages, at a detection level of 0.1% and prevalence level of 0.75%. Euler diagrams were generated for core microbiomes present in different generations; using the R package *Eulerr* package version 6.1.1 (Larsson, 2018). The differences in core bacterial communities across generations were analyzed by performing PERMANOVA on weighted and unweighted UniFrac distances.

## Results

The constructed amplicon 16S rRNA gene libraries generated 8,323,440 high-quality reads with an average coverage of 138,724 reads per sample. The reads were rarefied to 14,000 reads per sample (without replacement). Out of the 58 samples, one library with a number of reads below the cut-off was discarded. After normalization, we obtained 9535 ASVs, distributed among 27 phyla and 383 genera.

We first determined the relative abundance of the most abundant phyla and genera in the buccal cavity mucus and posterior intestine of the wild fish. Thereafter, to understand the microbial transfer across generations, we describe the differences in composition between two F0 families and the corresponding inbred F2, and between F0 and one outbred family (Figure 1). Later we disclose the differences in the bacteria between fish groups within F2.

### Microbial Composition in the Wild Parents, F0, and F2 Generations

#### Relative Abundance of Bacteria in Wild Fish

**FIGURE 2:**
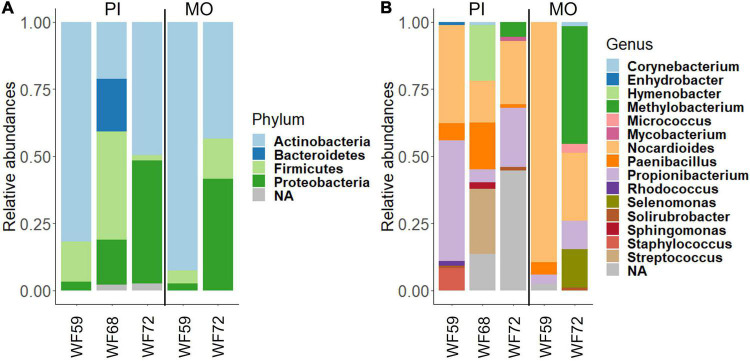
Relative abundance of bacteria found in the buccal cavity mucus and posterior intestine of wild Nile tilapia. **(A)** Phylum level. **(B)** Genus level. Three samples from the posterior intestine of three wild fish (WF59, WF68, and WF72). However, for the buccal cavity we employed the samples from only two wild fish (WF59 and WF72). MO, buccal cavity; PI, posterior intestine; NA, Unclassified.

The dominant phyla in the buccal cavity and posterior intestine of the wild fish were *Actinobacteria, Proteobacteria*, and *Firmicutes*. However, their abundance was different across samples (Figure 2A). The most abundant genera in the buccal cavity and posterior intestine were *Nocardioides*, *Propionibacterium*, *Paenibacillus*, and *Methylobacterium* (Figure 2B).

#### Relative Abundance of Bacteria in F0 and F2 Generations From Different Mothers

**FIGURE 3:**
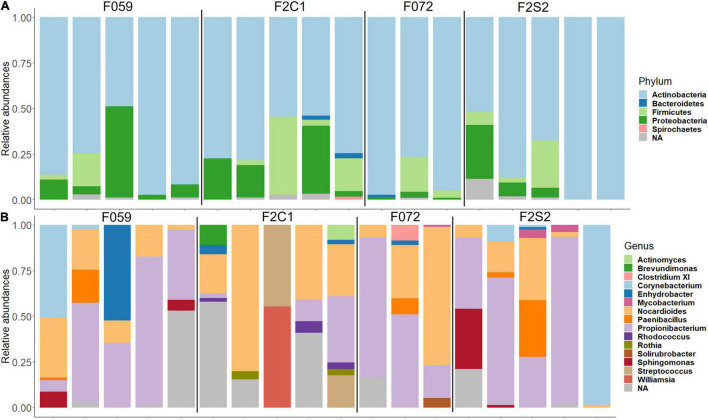
Relative abundance of bacteria found in the buccal cavity mucus of Nile tilapia bred in captivity. **(A)** Phylum level. **(B)** Genus level. F0 generation: F059, F072 and F2 generation: F2C1, F2S2.

**FIGURE 4:**
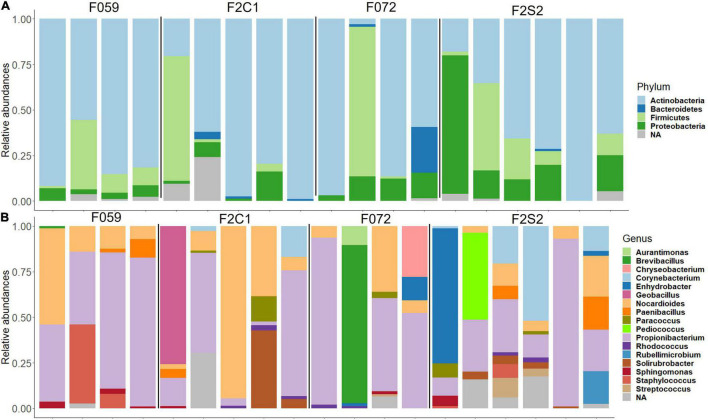
Relative abundance of bacteria found in the mucus of the posterior intestine from Nile tilapia bred in captivity. **(A)** Phylum level. **(B)** Genus level. F0 generation: F059, F072 and F2 generation: F2C1 (Inbred), F2S2 (Inbred). NA, Unclassified.

The most abundant phyla in the buccal cavity mucus in F0 and F2 generations were *Actinobacteria* followed by *Firmicutes*, *Proteobacteria*, and *Bacteriodetes* (Figure 3A). At the genus level, the buccal cavity was mostly dominated by *Propionibacterium* and *Nocardioides* (Figure 3B). However, other genera such as *Paenibacillus*, *Sphingomonas*, *Corynebacterium*, and *Enhydrobacter* were also common but in lower abundance compared to *Propionibacterium* and *Nocardioides* (Figure 3B). The most dominant phyla in the posterior intestine of the F0 and F2 generations were *Actinobacteria, Firmicutes*, and *Proteobacteria* (Figure 4A) while the dominant genera were *Propionibacterium*, *Nocardioides*, and *Solirubrobacter* (mostly in F2), *Corynebacterium*, *Enhydrobacter*, and *Paracoccus* (Figure 4B).

### Alpha and Beta Diversity and Differential Abundance Across Generations

**FIGURE 5:**
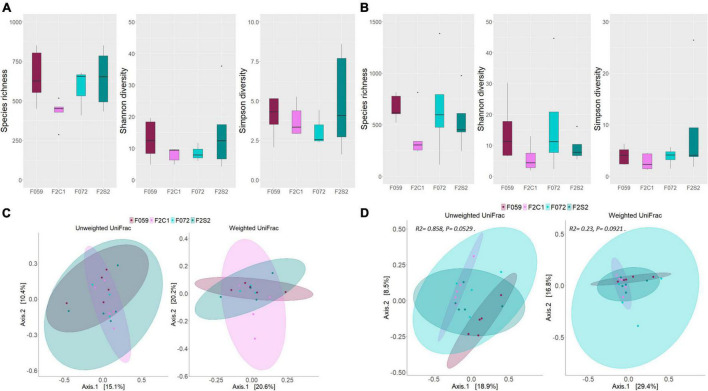
Differences in microbial diversity and composition of mucus bacteria from the buccal cavity and posterior intestine of Nile tilapia from F0 and F2 generations. Chao1, Shannon and Simpson diversities of **(A)** buccal cavity bacteria, **(B)** intestine bacteria. PCoA plots of the unweighted and weighted distances associated with **(C)** buccal cavity bacteria, **(D)** posterior intestine bacteria. Note that in panel **(C)**, the ellipse is not drawn for F072 because there were only three samples for this group. F0 generation: F059, F072 and F2 generation: F2C1, F2S2.

To delineate the alpha diversity of the bacterial communities in the mucus of the buccal cavity and posterior intestine of the F0 and F2 generations, we employed three different ecological diversity measures. Species richness (Chao1), effective number of common (Shannon diversity), and dominant bacteria (Simpson diversity) in the mucus of buccal cavity as well as posterior intestine were not significantly different between F0 and F2 generations (Figures 5A,B). Weighted and unweighted UniFrac distances-based beta diversity analysis also did not reveal any difference between the mucus bacterial communities (both from the buccal cavity and posterior intestine) of the different generations (Figures 5C,D). As for the posterior intestine of the fish families, we observed a statistical trend that indicated the difference in the weighted and unweighted UniFrac distances of the microbial communities [Figure 5D: unweighted UniFrac (F059, F2C1, F072, and F2S2); *R*^2^ = 0.86, *P* = 0.05, Figure 5D: weighted UniFrac (F059, F2C1, F072, and F2S2); *R*^2^ = 0.23, *P* = 0.09]. The *post-hoc* test revealed differences in the microbial communities in the posterior intestine between F059 (F0) and F2C1 (F2) (unweighted UniFrac; P = 0.04). Although F059 and F2C1 are statistically significant, DESeq2 did not find any ASV that is significantly different between the two fish groups.

### Core Microbiome in the Wild Fish and Two Generations Bred in Captivity

**FIGURE 6:**
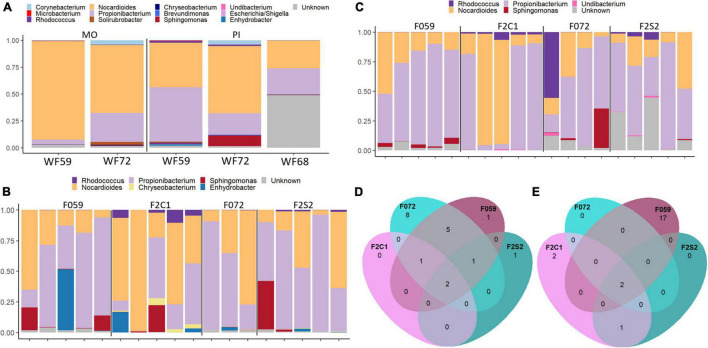
Core and shared microbiome in the mucus from the buccal cavity and posterior intestine of Nile tilapia from wild, F0, and F2 generations. **(A)** Core microbiome in the mouth mucus and posterior intestine of wild Nile tilapia. Core microbiome in the **(B)** buccal cavity and **(C)** intestine of F0 and F2 generations. Shared core microbiome in the **(D)** buccal cavity and **(E)** intestine of F0 and F2 generations. In panel **(D)**, five ASVs of *Propionibacterium* were common in both F059 and F072 and one ASV was common in both F0 and F2 generations. MO, buccal cavity; PI, posterior intestine. F0 generation: F059, F072 and F2 generation: F2C1, F2S2.

To investigate the microbial transfer across generations, first we identified the core microbiome/microbes (present in 80% of the samples) in the wild fish. We presume that these microbes are essential for the host and therefore are transferred from one generation to another or common between generations. Hence, we also identified the shared core microbiome that is found in both F0 and F2 generations. At the genus level, *Nocardioides* and *Propionibacterium* were the most abundant core microbiome members in the mucus from the buccal cavity and posterior intestine of the wild fish (WF59, WF68, and WF72) (Figure 6A). Moreover, *Sphingomonas* and *Corynebacterium* were also core microbiome members in both the mouth and posterior intestine (Figure 6A). The buccal cavity mucus of F0 and F2 generations also had both *Nocardioides* and *Propionibacterium* as the most abundant members of the core microbiome (Figure 6B). Furthermore, *Sphingomonas* and *Enhydrobacter* were also abundant in some fish from F0 and F2 generations (Figure 6B). In addition, we found *Rhodococcus* in low abundance in the F2 generation. Furthermore, one ASV which is classified as *Propionibacterium* was common in both F0 and F2 generations, while three ASVs that also belong to the genus *Propionibacterium* were common only in the F0 generation (F059 and F072). ASVs of *Actinomycetales* were also shared in the F0 generation. Moreover, two ASVs of the genus *Nocardioides* were common in F0 and F2 generations (Figure 6D and Supplementary Table 1). In the posterior intestine of all the samples from both lineages (F059, F2C1, and F072, F2S2), *Nocardioides* and *Propionibacterium* were the most abundant bacteria (Figure 6C). Furthermore, in F0 (F059 and F072) *Sphingomonas* was also observed as the prominent genus, but not detected as frequently as *Nocardioides* and *Propionibacterium.* Moreover, ASVs of *Nocardioides* (*DENOVO* 2) and *Propionibacterium* (*DENOVO* 1) were present in different generations (Supplementary Table 2 and Figure 6E). However, one ASV belonging to *Nocardioides* (*DENOVO* 2) was not found in F2S1 generation.

### Comparison of the Microbial Composition Among Second Generation Families of Nile Tilapia

The differences/similarities in the buccal cavity and posterior intestine mucus of families F2C1, F2S1, and F2S2 from the second generation of Nile tilapia were studied. In the buccal cavity of F2C1 and F2S2, the most dominant phyla were *Actinobacteria, Proteobacteria, Firmicutes*, *Bacteroidetes*, and *Spirochaetes*; latter two only in some samples (Figure 3A). At the genus level, *Propionibacterium*, *Nocardioides*, and *Corynebacterium* (in some samples) were the most abundant bacteria in both families (Figure 3B). Furthermore, in the F2C1 family, *Rhodococcus* and *Enhydrobacter* were also abundant in some samples. On the other hand, in F2S2 family, *Sphingomonas* appeared in some of the samples. In the posterior intestine, *Actinobacteria, Firmicutes, Proteobacteria*, and *Bacteroidetes* were the most dominant phyla in both families (Figure 4A). In the F2S2 family, in addition to *Propionibacterium, Nocardioides* and *Corynebacterium, Paenibacillus* (in some samples), and *Pediococcus* (in some samples) also belonged to the most dominant genera (Figure 4). On the other hand, the buccal cavity of F2C1 and F2S1 were mostly dominated by *Actinobacteria, Firmicutes, Proteobacteria, Bacteroidetes, Nitrospirae* (F2S1, one sample), and *Spirochaetes* (F2C1, one sample). Bacteria belonging to *Nitrospirae* were more dominant in F2S1, while *Spirochaetes* were higher in F2C1 (Supplementary Figure 1A). The most abundant genera in the buccal cavity were *Propionibacterium* and *Nocardioides*. The F2S1 family was mostly dominated by *Propionibacterium*. In addition, *Nocardioides, Sphingomonas, Spirosoma*, and *Nitrospira* were also abundant in some samples of F2S1 (Supplementary Figure 1B). In the posterior intestine of F2C1 and F2S1, *Actinobacteria, Firmicutes, Proteobacteria*, and *Bacteroidetes* were dominant (Supplementary Figure 2A). At the genus level, F2C1 was mostly dominated by *Propionibacterium, Nocardioides, Paracoccus*, and *Solirubrobacter.* The F2S1 family was dominated by *Propionibacterium* (Supplementary Figure 2B) but *Sphingomonas, Geobacillus, Paenibacillus*, and *Alloiococcus* were also abundant.

### Alpha and Beta Diversity and Differentially Abundant Amplicon Sequence Variants in the F2 Families From the Second Generation of Nile Tilapia

Statistically significant differences were not detected for the alpha diversity measures of the mucus bacterial communities (in the buccal cavity as well as posterior intestine) between F2C1 and F2S2 families (Figures 5A,B). Similarly, beta diversity analysis did not reveal any statistically significant differences in both unweighted and weighted UniFrac distances (Figures 5C,D). Statistically significant differences were also not found in alpha diversity values of the F2C1 and F2S1 families (Supplementary Figures 3A,B). However, statistically significant differences were detected for the weighted UniFrac distances; for both the buccal cavity and the posterior intestine microbiota (*R*^2^ = 0.67, *P* = 0.01; *R*^2^ = 0.30, *P* = 0.02, respectively; Supplementary Figures 3C,D).

We performed differential abundance analyses to understand the differences between the buccal cavity bacteria of inbred families (F2S2 vs. F2C1) and between outbred and inbred (F2S1 vs. F2S2) to investigate the influence of breeding strategy on the buccal cavity microbial composition. The result revealed that one ASV had significantly lower abundance in F2S2 compared to the F2C1: *Kocuria* with a log2foldchange (LFC) of −35 (Supplementary Figure 4A). On the other hand, in the buccal cavity of F2S1, *Corynebacterium*, *Propionibacterium*, and *Staphylococcus* had higher abundance (more than 5 LFC) compared to the F2C1 family (Supplementary Figure 4B). While the abundance of *Nocardioides* and *Rothia* were lower (5 LFC) in F2S1 compared to F2C1 (Supplementary Figure 4B). In the case of the posterior intestine, *Corynebacterium* had lower abundance (25 LFC) in F2S1 compared to F2C1. Furthermore, *Brevibacillus, Geobacillus, Paenibacillus*, and *Sphingomonas* had higher abundance (10 LFC) in the F2S1 family compared to F2C1 (Supplementary Figure 4C).

## Discussion

Microbial transmission from different body sites of parents to offspring is extensively studied in humans compared to other animals. The gathered data on microbial transmission have provided insights into the beneficial as well as disease-causing microbes that are passed on to generations. A recent study has reported that bacterial transmission is dependent on both relationship and cohabitation (Valles-Colomer et al., 2022). Transfer of bacteria from mother to infant shapes the microbial composition in infants. It should be noted that delivery mode (cesarean section) can disrupt the normal assemblage of microbes in infants, and this issue can make the offspring susceptible to diseases such as celiac disease, asthma, and obesity (Mueller et al., 2015). These facts indicate the importance of normal microbial community at the early stage of organism development. Only a few studies have reported microbe transfer in aquatic animals. These studies showed evidence of vertical microbial transmission (Stephens et al., 2016; Sylvain and Derome, 2017). Here we present the first report of bacterial transmission across generations of Nile tilapia, a mouthbrooder fish species.

Our results revealed the dominance of the phyla, *Actinobacteria, Proteobacteria*, and *Firmicutes* in the buccal cavity and posterior intestine of the wild Nile tilapia individuals. These phyla are known to be dominant in the gut of wild Nile tilapia (Bereded et al., 2020; Bereded et al., 2021) and many other fish species (Legrand et al., 2020; Yukgehnaish et al., 2020). On the other hand, the buccal cavity microbiome of wild Nile tilapia has not been previously reported. The microbial composition in the buccal cavity and the posterior intestine in F0 and F2 generations was also dominated by the aforementioned phyla. Furthermore, *Actinobacteria, Proteobacteria*, and *Firmicutes* were found as the dominant phyla in breast tissue and human milk (Togo et al., 2019). In our study, at the genus level, the most abundant bacteria were *Propionibacterium* and *Nocardioides*; in both the buccal cavity and the posterior intestine. We found that these genera are also dominant in the wild fish samples though their abundance was different across individuals.

Various body sites of fishes harbor microbes and different factors such as diet, environment, and host pressure may help in the establishment of a balanced healthy microbiota which is known as normobiosis (Johny et al., 2021). From an ecological point of view, niche- and neutral- processes lead to well-established host microbial communities (Liao et al., 2016). The niche-based theory indicates the deterministic effects of factors such as environmental conditions, among which rearing systems can influence the gut microbiome assemblage during the development of Nile tilapia larvae. Shared OTUs of the rearing water and gut bacterial communities of Nile tilapia larvae points to the niche selection of the water bacteria (Giatsis et al., 2015). Distinct core gut microbiota in zebrafish was suggested to be due to host selective pressure or a niche selection based on certain bacteria in the rearing water (Roeselers et al., 2011). A study reported differences in the gut of larvae reared in two different rearing systems. However, when they were moved to a common recirculating aquaculture system (RAS), the gut microbial diversity and composition was similar in the individuals (Giatsis et al., 2014; Deng et al., 2021). It is also known that as fishes grow host pressure overtakes the environmental factors in deciding the microbial profile (Talwar et al., 2018). In the present study, we did not find any difference in the microbial richness and evenness in F0 and F2 generations that were reared in a common garden. This may indicate that the oral microbiome is colonized by similar microbial communities. However, in the case of the posterior intestine, we found a statistical trend, probably indicating a difference between the microbial communities in F0 and F2 generations. We speculate that the differences are due to breeding/genetic effects in F059 (F0) and F2C1 (F2) families (Abdelhafiz et al., 2021a). To understand this fact, we analyzed the microbial composition in two families of the F2 generation; these results also did not reveal any differences in the diversities of the microbial communities in F2C1 and F2S2, which are both inbred groups. On the other hand, we found dissimilarities between the microbial community compositions of the buccal cavity and the posterior intestine in F2C1 (inbred) and F2S1 (outbred), based on the weighted UniFrac distance of the inbred and outbred groups. Furthermore, the differential expression analysis of ASVs revealed significant differences between ASVs in both the buccal cavity and the posterior intestine in all the F2 family comparisons. When the buccal cavity communities of the two inbred groups (F2C1 and F2S2) were compared, *Kocuria* was noted to be the less abundant bacteria in F2S2 compared to F2C1. On the other hand, when we compared the buccal cavity communities of an inbred group with those of an outbred group (F2C1 and F2S1, respectively) we found differences in the microbial taxa. Furthermore, one of the ASVs of the core microbiome belonged to *Nocardioides* (*DENOVO* 2), which was not found in the outbred group (F2S1). The differences between the microbial communities, in this case, are likely due to the breeding strategy (Abdelhafiz et al., 2021a).

The core microbiome is known to be present across any population of a particular host organism, and this community plays an essential role in the host biological functions (Risely, 2020). Although it is well known that many factors modulate the microbiome composition in a host, the presence of the core microbial community may not be disrupted (Salonen et al., 2012; Henderson et al., 2015; Deng et al., 2021). In our previous study, we reported the lower microbial inter-individual variability amongst the intestine bacteria of the inbred Nile tilapia (Abdelhafiz et al., 2021a). However, in the present study, our analysis showed inter-individual variation across the buccal cavity and posterior intestine samples of the F2 and F0 generation. In fish, microbial inter-individual variation is common. This was observed even in individuals (cod and bluefin tuna larvae) reared in the same tank (Fjellheim et al., 2012; Gatesoupe et al., 2013). Furthermore, when the microbial interactions between core microbiome members and other microbes are positive, competition between the microbes will be less (Jones et al., 2018), allowing host genetic/selection or ecological pressure to shape the microbial compositional variation (Shan and Cordero, 2020). Moreover, the inter- and intra-individual compositional variations in humans are regarded stable over time (Jones et al., 2018).

In Nile tilapia larvae, the core microbiome was not affected by the early life environment (Deng et al., 2021) and these bacteria had high abundance (Wu et al., 2020; Deng et al., 2021). In the current study, the abundance of the members of the core microbiome was high in the buccal cavity and the intestine of the wild as well as F0 and F2 generations, mostly dominated by *Nocardioides, Propionibacterium, Sphingomonas*, and *Enhydrobacter*. However, the core microbiome in the intestine of wild Nile tilapia from Lake Awassa and Chamo in Ethiopia was reported by Bereded et al. (2020). At the phylum level, the core microbiome in the fishes from these two lakes was similar to wild tilapia (in the current study) from the Nile river in Egypt. The core microbiome was mostly dominated by *Actinobacteria, Firmicutes*, and *Proteobacteria.* However, at the genus level, the core microbiome in our study and that of Bereded et al. (2020) were different. In Awassa and Chamo lakes, the most abundant genera were *Clostridium_XI, GPXI, Cetobacterium*, and *Turicibacter.* In the current study, the core microbiome was mostly dominated by *Nocardioides, Propionibacterium, Sphingomonas*, and *Corynebacterium.* In our previous study, we observed *Cetobacterium* as a core member in the mouth and intestine of Nile tilapia from the F1 generation (Abdelhafiz et al., 2021a). These differences in the core microbiome could be attributed to the microbial functional groups. It was reported that microbes with similar metabolic functions can be combined into functional groups which are controlled by various ecological pressures (Shan and Cordero, 2020). Furthermore, in blue tilapia (*Oreochromis aureus*) maternal cold-tolerant genetic components were reported to be transferred to offspring (Nitzan et al., 2016). In addition, Kokou et al. (2018) reported host-microbe selection of cold-tolerant microbes in the gut of blue tilapia. Therefore, we also speculate that the difference in the core microbiome between wild Nile tilapia from Egypt and Ethiopia could be due to a genetic pressure directed toward environmental factors.

The core microbiome that is vertically transmitted across generations (Funkhouser and Bordenstein, 2013; Sylvain and Derome, 2017; Lee et al., 2019; Jorge et al., 2020) has conserved functions (Ramos et al., 2021). In the current study, we observed a presumed vertical transmission of the core microbiome from the wild Nile tilapia to the subsequent generations (F0 and F2). The core microbiome in the buccal cavity was mostly dominated by different ASVs of *Nocardioides, Propionibacterium, Sphingomonas*, and *Enhydrobacter.* These core members were also abundant in the posterior intestine, the exception was *Enhydrobacter*. Breast milk microbiome of humans is dominated by nine genera, and among them are *Propionibacterium* and *Sphingomonas* (Mueller et al., 2015). In infants, breastfeeding promotes the colonization and maturation of the infant gut microbiome in addition to the vertically transmitted microbes from different body sites of mothers (Mueller et al., 2015). However, microbes transmitted from the mother’s skin and vagina are transient microbes that facilitate the early colonization of other microbes also. Maternal gut microbes that are known to have better ecological adaptation capacity were found to be more persistent in the infant gut (Ferretti et al., 2018). In discus fish, maternal skin microbiome that is vertically transmitted to offspring shapes the gut microbial community of the fry (Sylvain and Derome, 2017). In our study, we found that microbes from both maternal mouth and gut shape the microbiome in offspring. For example, in wild fish, *Sphingomonas* was a member of the core microbiome of only posterior intestine samples. Nevertheless, we detected bacteria belonging to this genus in the mouth of F0 and F2 generations. The egg capsule of little skate was reported to have a high microbial richness and core microbiome that are essential for embryonic development (Mika et al., 2021). Therefore, we speculate that *Nocardioides, Propionibacterium*, and *Sphingomonas* may have a role in facilitating the colonization of other microbes in the buccal cavity and gut of Nile tilapia. Furthermore, the incubation of eggs in the buccal cavity of Nile tilapia could be the route for vertical transmission of microbes to the eggs. *Propionibacterium* have been found in human skin microbiome, raw milk, soil, silage, and anaerobic digesters (Gautier, 2014). Moreover, members of *Propionibacterium* were reported to break down urea and release ammonia (Gautier, 2014). It was reported that carp and zebrafish gill nitrogen-cycle microbes can detoxify ammonia (van Kessel et al., 2016). In European seabass, *Propionibacterium* was noted to be dominant in digesta and mucosa (Serra et al., 2021). Furthermore, *Propionibacterium* species are known for their unique metabolism to convert lactate to propionic acid and acetic acid by fermentation (Ciani et al., 2013). In addition, *Propionibacterium* sp. have immunomodulatory effects in the mice intestine. It was reported that propionate, which is produced by *Propionibacterium*, prevents acute colitis in mice (Plé et al., 2015). It is also known that *Propionibacterium* surface proteins interact with human epithelial cell surface and improve barrier functions in the intestine, and probably act against inflammatory bowel diseases (do Carmo et al., 2017). *Nocardioides*, the other abundant member of the core microbiome in Nile tilapia, are known to produce the anti-tumor antibiotics, sandramycin, and they can modify complex compounds chemically and enzymatically. Moreover, *Nocardioides* spp. have antimicrobial and antifungal activities (Lee et al., 2012), and they belong to healthy microbiota, as reported in the case of feces from healthy cottontail rabbits (Zhang et al., 2015). Furthermore, bacteria from this genus were found enriched in mice intestine after *Lactobacillus plantarum* administration (Xie et al., 2016). *Nocardioides* was also reported as gut microbiome member in Malaysian population (Chua et al., 2019). Other articles have also indicated the presence of *Nocardioides* and *Sphingomonas* in other fishes and fish rearing facilities; *Nocardioides* in the intestine of Korean spotted sleeper (*Odontobutis interrupta*) and leopard mandarin fish (*Siniperca scherzeri*) (Hyun et al., 2021), and *Sphingomonas* in fish ponds (Chen et al., 2016) and fish intestine (Hyun et al., 2021).

*Sphingomonas* species produce poly-β-hydroxybutyrate, in situations where carbon is available, but when there is a limited nutrient source (Dedkova and Blatter, 2014; Chen et al., 2016). Furthermore, *Sphingomonas* species can grow and survive in a wide range of environments that other bacteria do not tolerate (Kuehn et al., 2013). *Sphingomonas* was found as a core microbiome member in healthy human milk (Hunt et al., 2011), in Cyprus donkey milk (Papademas et al., 2021) and in bovine milk (Kuehn et al., 2013). In humans, feces microbiome of infants are different between breastfed and formula-fed infants, which indicates the transfer of microbes from milk to infant gut and/or involvement of milk prebiotics in the proliferation of specific microbes (Moossavi et al., 2018). Disturbance of the transfer process of the microbes from human milk to the infant was associated with many diseases (Hunt et al., 2011). *Sphingomonas* protect maternal breast tissue against breast cancer, and the abundance of *Sphingomonadaceae* family was higher in the nipple aspirate fluid of healthy women (Xuan et al., 2014; Chan et al., 2016). Thus, *Propionibacterium, Nocardioides*, and *Sphingomonas* that are transmitted vertically from the wild fish and possess the aforementioned functional potential are likely beneficial members in the core microbiome of Nile tilapia.

## Conclusion

Here we report for the first time a presumed vertical transmission (based on similar ASVs in different generations) of buccal cavity and intestine microbial communities across generations in a mouthbrooder species. To our knowledge, the buccal cavity microbiomes in wild Nile tilapia has not been previously reported. We presume that the buccal cavity and the intestine core microbiome facilitate the colonization of other gut microbiome across generations. Furthermore, we suggest that the route of vertical transmission is through the mouth when eggs are incubated in the buccal cavity of Nile tilapia. Based on the literature, we believe that the core microbiome members that were likely vertically transmitted from the wild tilapia are beneficial bacteria and could play an essential role in the development of the offspring.

## Data Availability Statement

The data presented in this study are deposited in the repository, Sequence Read Archive (SRA) with accession number PRJNA808265.

## Ethics Statement

The animal study was reviewed and approved by this study was performed under a license from the Norwegian Animal Research Authority (FOTS ID 10427).

## Author Contributions

VK, JF, and YA designed the study. YA carried out the sampling, lab work, analyzed the data, and wrote the manuscript. CD also analyzed the data. MP performed sequencing and data generation. YA, JF, CD, and VK interpreted the data. VK, JF, and CD reviewed and edited the manuscript. All authors approved the manuscript.

## Conflict of Interest

The authors declare that the research was conducted in the absence of any commercial or financial relationships that could be construed as a potential conflict of interest.

## Publisher’s Note

All claims expressed in this article are solely those of the authors and do not necessarily represent those of their affiliated organizations, or those of the publisher, the editors and the reviewers. Any product that may be evaluated in this article, or claim that may be made by its manufacturer, is not guaranteed or endorsed by the publisher.
