# Supplementary material for: Intergenerational Transfer of Persistent Bacterial Communities in Female Nile Tilapia
Source: Front Microbiol. 2022 May 17;13:879990. doi: 10.3389/fmicb.2022.879990 (PMC9152445; doi:10.3389/fmicb.2022.879990)
Supplement: Supplementary file 1 [file Data_Sheet_1.docx]

**Intergenerational transfer of persistent communities in female Nile tilapia**

Yousri Abdelhafiz^1^, Jorge M. O. Fernandes^1^, Claudio Donati^2^, Massimo Pindo^2^, Viswanath Kiron1*

^1^Faculty of Biosciences and Aquaculture, Nord University, Norway

^2^ Unit of Computational Biology, Research and Innovation Centre, Fondazione Edmund Mach, Via E. Mach 1, 38098, San Michele all'Adige, Italy


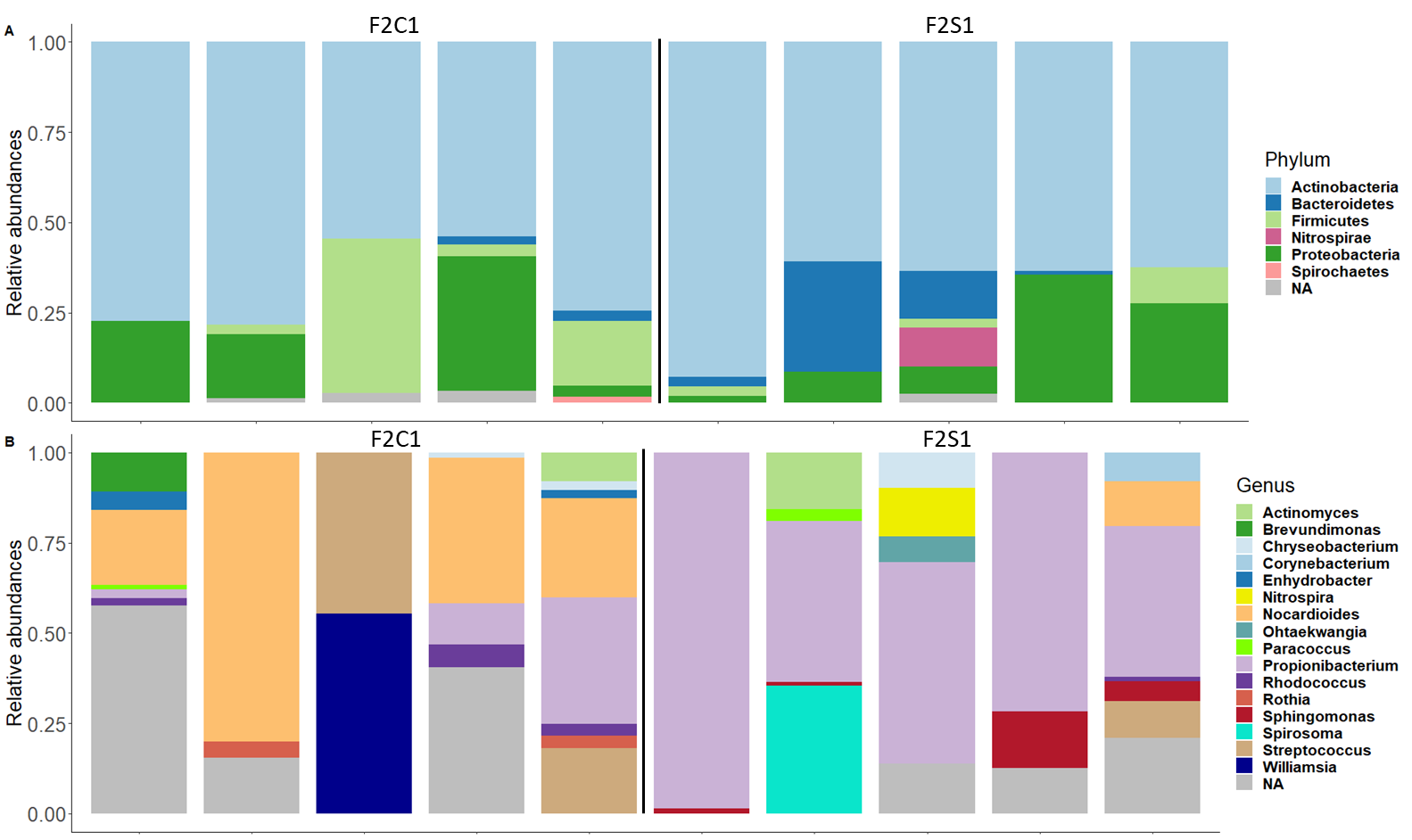


**Supplementary figure 1. Relative abundance of mucus bacteria in the buccal cavity of two families from second (F2) generation (F2C1; inbred and F2S1; outbred) of Nile tilapia.** A) Phylum level. B) Genus level. NA-Unclassified.


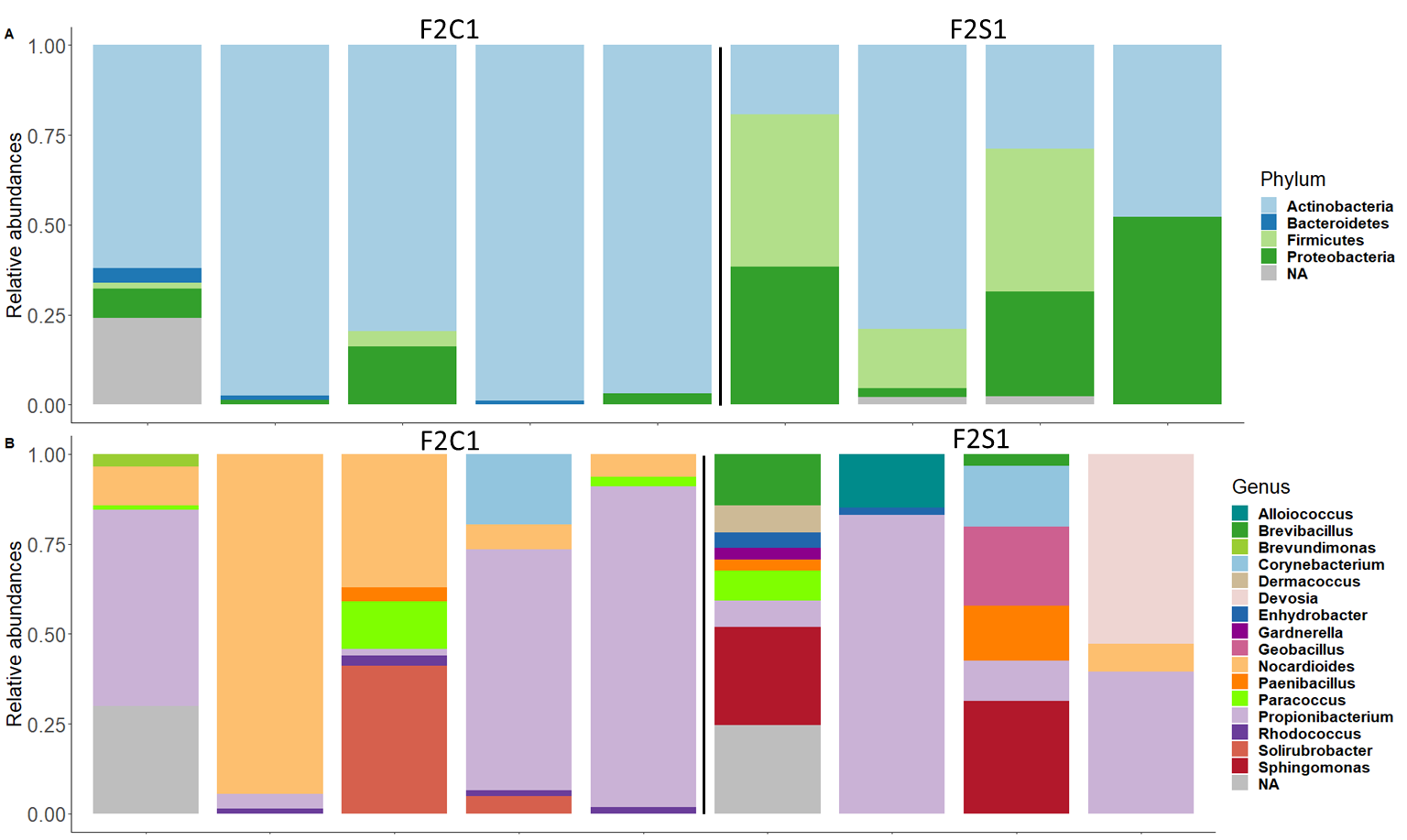


**Supplementary figure 2. Relative abundance of mucus bacteria in the posterior intestine of two families from outbred second (F2) generation (F2C1; inbred and F2S1; outbred) of Nile tilapia.** A) Phylum level. B) Genus level. NA-Unclassified.


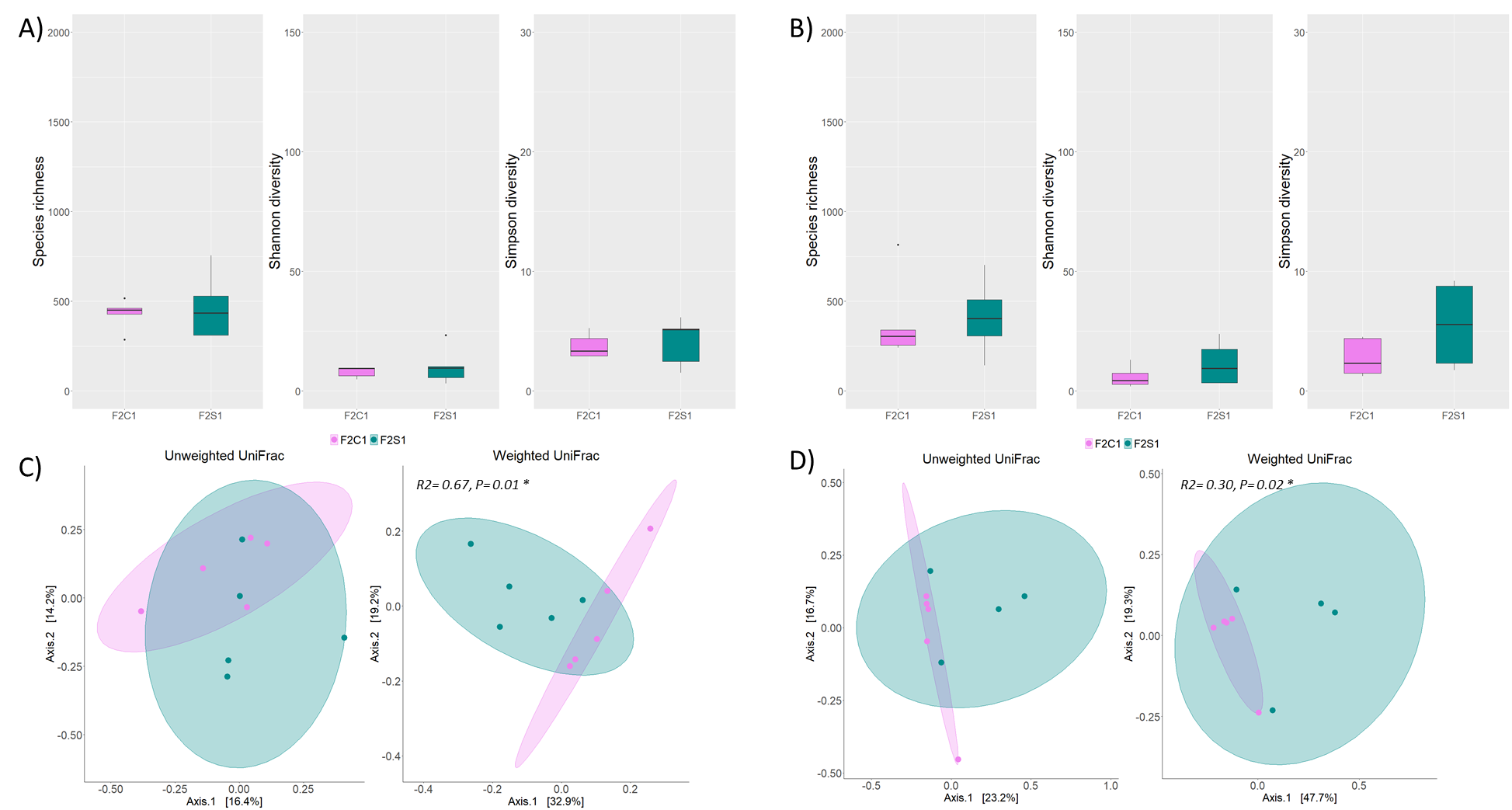


**Supplementary figure 3. Differences in microbial diversity and composition of mucus bacteria from the buccal cavity and posterior intestine of Nile tilapia from families in F2 generations (F2C1; inbred and F2S1; outbred).** Chao1, Shannon and Simpson diversities of the A) buccal cavity bacteria, B) intestine bacteria. PCoA plots of the unweighted and weighted distances associated with the C) buccal cavity bacteria, D) intestine bacteria.


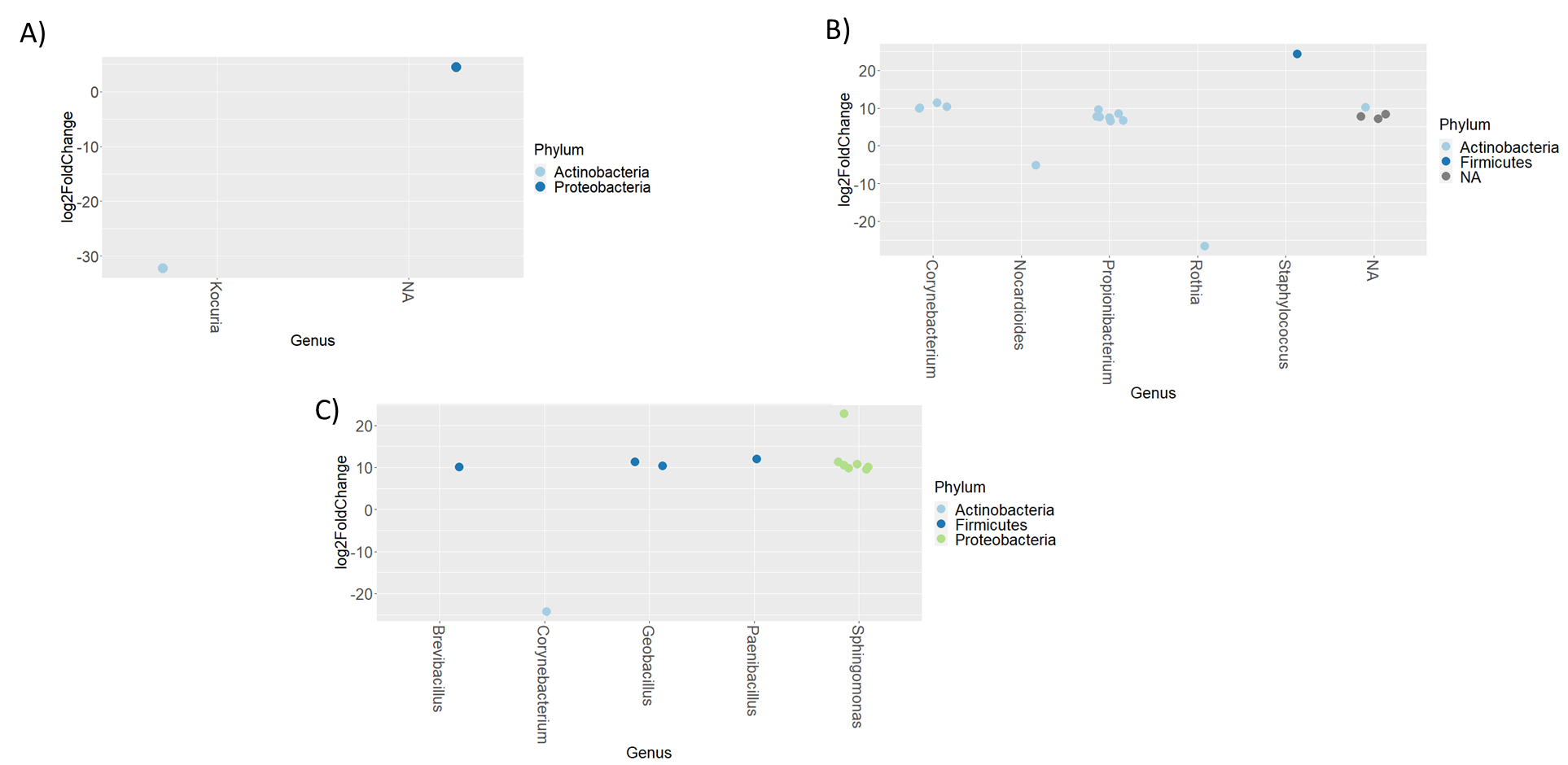


**Supplementary figure 4. Differentially abundant ASVs between families in F2 generation.** A) In the buccal cavity from F2S2 (inbred) vs F2C1 (inbred) comparison. B) In the buccal cavity from F2S1 (outbred) vs F2C1 (inbred) comparison. C) In the posterior intestine from F2S1 (outbred) vs F2C1 (inbred) comparison.

**Supplementary Table 1. Buccal cavity core microbiome that was shared between families and generations**

| **ASV** | **Genus** | **Body site** | **Family and generation** |
| --- | --- | --- | --- |
| DENOVO95 | *Nocardiodies* | MO | F059, F072 and F2C1 |
| DENOVO2 | *Nocardiodies* | MO | F059, F072, F2C1 and F2S2 |
| DENOVO1 | *Propionibacterium* | MO | F059, F072, F2C1 and F2S2 |
| DENOVO6 | *o__Actinomycetales* | MO | F2S2 |
| DENOVO55 | *o__Actinomycetales* | MO | F059, F072 and F2S2 |
| DENOVO98 | *o__Actinomycetales* | MO | F072 |
| DENOVO111 | *o__Actinomycetales* | MO | F059 and F072 |
| DENOVO123 | *Nocardiodies* | MO | F072 |
| DENOVO125 | *Propionibacterium* | MO | F072 |
| DENOVO61 | *o__Actinomycetales* | MO | F059 and F072 |
| DENOVO138 | *Propionibacterium* | MO | F072 |
| DENOVO73 | *Propionibacterium* | MO | F059 and F072 |
| DENOVO124 | *o__Actinomycetales* | MO | F072 |
| DENOVO126 | *Propionibacterium* | MO | F072 |
| DENOVO63 | *Nocardiodies* | MO | F072 |
| DENOVO118 | *Propionibacterium* | MO | F059 and F072 |
| DENOVO8 | *Sphingomonas* | MO | F059 |
| DENOVO31 | *Propionibacterium* | MO | F059 and F072 |
| DENOVO89 | *Nocardiodies* | MO | F072 |

MO- Mouth

**Supplementary Table 2**. Core microbiome that were shared across generations

| **ASV** | **Genus** | **Body site** | **Family and generation** |
| --- | --- | --- | --- |
| DENOVO95 | *Nocardiodies* | MO | F059, F072 and F2C1 |
| DENOVO2 | *Nocardiodies* | MO and PI | All generations including wild fish except F2S1 |
| DENOVO1 | *Propionibacterium* | MO and PI | All generations including wild fish |
| DENOVO73 | *Propionibacterium* | MO | WF59, F059 and F072 |
| DENOVO31 | *Propionibacterium* | MO | WF59,WF72, F059 and F072 |
| DENOVO118 | *Propionibacterium* | MO | F059 and F072 |
| DENOVO126 | *Propionibacterium* | PI | WF59 and F059 |
| DENOVO123 | *Nocardiodies* | PI | WF59 and F059 |
| DENOVO118 | *Propionibacterium* | PI | WF59 and F059 |
| DENOVO95 | *Nocardiodies* | PI | WF59 and F059 |
| DENOVO37 | *Propionibacterium* | PI | WF59 and F059 |
| DENOVO8 | *Sphingomonas* | PI | WF59 and F059 |
| DENOVO138 | *Propionibacterium* | PI | WF59 and F059 |
| DENOVO63 | *Nocardiodies* | PI | WF59 and F059 |
| DENOVO31 | *Propionibacterium* | PI | WF59 and F059 |
| DENOVO89 | *Nocardiodies* | PI | WF59 and F059 |

MO- Mouth, PI- Posterior intestine
